# Non-Instrumental and Instrumental Tools Validity in Bruxism Diagnostics

**DOI:** 10.3390/diagnostics15020200

**Published:** 2025-01-16

**Authors:** Adrian Marcel Popescu, Mihaela Ionescu, Diana Elena Vlăduțu, Sanda Mihaela Popescu, Iulia Roxana Marinescu, Monica Scrieciu, Veronica Mercuț

**Affiliations:** 1 Department of Dental Prosthetics, Faculty of Dentistry, University of Medicine and Pharmacy of Craiova, 200349 Craiova, Romania; smpopescu@mail.com (A.M.P.); monica.scrieciu@umfcv.ro (M.S.); veronica.mercut@umfcv.ro (V.M.); 2 Department of Medical Informatics and Biostatistics, Faculty of Dentistry, University of Medicine and Pharmacy of Craiova, 200349 Craiova, Romania; 3 Department of Oral Rehabilitation, Faculty of Dentistry, University of Medicine and Pharmacy of Craiova, 200349 Craiova, Romania; sanda.popescu@umfcv.ro (S.M.P.); roxana.marinescu@umfcv.ro (I.R.M.)

**Keywords:** bruxism diagnostic, questionnaire, clinical exam, clenching, grinding, sEMG, stress, anxiety, dental wear, masticatory muscles activity

## Abstract

**Background/Objectives:** The study aimed to validate the diagnostic system proposed by the Standardized Tool for the Assessment of Bruxism (STAB) by correlating the results obtained based on questionnaire and non-instrumental and instrumental tools. **Methods:** The study had three stages (questionnaire, clinical examination, and electromyographic study). The subjects completed a questionnaire and clinical exam. Positive signs of bruxism included oral mucosal signs and the presence of dental wear according to the BEWE index. In stage three, sEMG was performed after allocating subjects into four groups according to the questionnaire and clinical exam results: sleep bruxism (SB), awake bruxism (AB), sleep and awake bruxism (SB AB), and no bruxism (no B). After the third stage, a new selection was made, and the subjects were divided into four groups, according to sEMG results. Diagnostic accuracy was computed for possible bruxism SB and grinding and clenching sound diagnosis, possible bruxism AB and AB acknowledgment, possible bruxism SB AB, and tooth wear index. **Results:** For SB, the sensitivity and specificity of the tools were the highest. The non-instrumental questionnaire and clinical assessment identified 67% of SB cases and 89% without SB. For AB, the specificity was higher (84%), while the sensitivity was lower (55%), as almost half of the subjects were not aware of the presence of AB. The tests showed a low sensitivity (15%) but a high specificity (83%) for tooth wear. The absence of tooth wear was frequently associated with the absence of bruxism, while the presence of tooth wear did not necessarily imply the existence of bruxism. **Conclusions:** Non-instrumental evaluation of bruxism through questionnaires and clinical exams is valuable, especially for SB. Instrumental evaluation through electromyography remains a gold standard for bruxism diagnosis.

## 1. Introduction

Bruxism is a motor behavior of the masticatory muscles [1] whose effects on the masticatory system are important [2], leading to tooth wear [3,4], tooth fractures [5,6], pain of the masticatory muscles [7] and temporomandibular dysfunction [8]. The diagnosis of bruxism and its association with tooth wear have been the subject of much discussion over time [1,9,10], both for [11] and against [12]. In recent years, researchers worked to create a system (Standardized Tool for the Assessment of Bruxism or STAB) that integrates data obtained from medical history, clinical examination, and complementary/instrumental examinations [13,14]. As a result, a certain diagnosis of bruxism can be made, bruxism can be managed, and adverse effects of bruxism on the masticatory system are avoided [13,15]. The definition of bruxism has evolved in the last decade due to two international consensus papers published in 2013 and 2018 [1,9,16]. The latest consensus considers that sleep bruxism should be separated from awake bruxism based on different etiologies and manifestations, and individual definitions should be used. Thus, sleep bruxism (SB) is defined as an activity of the masticatory muscles during sleep characterized as rhythmic (phasic) or non-rhythmic (tonic) activity and is not a movement disorder or sleep disorder in healthy individuals. Awake bruxism (AB) is defined as an activity of the masticatory muscles during wakefulness characterized by sustained or repetitive tooth contact and/or bracing or thrusting of the mandible and is not a movement disorder in healthy individuals [1].

According to the second consensus, bruxism should no longer be considered a disease, condition, or disorder but a motor behavior with a multifactorial etiology [1]. Therefore, it can be an involuntary (sleep bruxism) or a conscious behavior (awake bruxism). Bruxism can have three connotations: harmless behavior (without consequences), risk factor (bruxism associated with one or more health problems), or protective factor (bruxism associated with one or more positive aspects of health) [1]. In any event, bruxism affects the oral cavity and can have consequences like tooth destruction, headaches, orofacial pain or temporomandibular dysfunction, and oral mucosa lesions produced by biting [10]. Therefore, bruxism is a risk factor for oro-dental diseases [17,18]. The following factors may exacerbate bruxism: stress [19,20], smoking [21,22], type A anxious personality [10,23], and sleep disorders, such as snoring [10,24]. Approximately 60–80% of sleep bruxism episodes are associated with restless legs syndrome [25,26].

Regarding prevalence, data vary for sleep and awake bruxism. The prevalence of bruxism varies according to the evaluation method but also depends on the continent, type of bruxism, and study type [27,28]. The last review of prevalence reports a global value of 22.22% for bruxism in general, estimating that one in four individuals is affected [27]. In Europe, sleep bruxism prevalence was 21% and awake bruxism 18%, with women being the most affected [27]. Other reported values for SB include an average of 16.5%, with a range between 8.31% and 21% [29]. In Romania, in a study conducted in 2021 on young dental students and published in 2022, the average prevalence of SB was 16.28% and of AB was 68.99%, while 14.73% presented a combined form of bruxism [19].

Lavigne recommended that for young, healthy subjects, the final diagnosis of moderate to severe sleep bruxism should be based on the following criteria: (i) the presence of a frequent grinding noise during sleep for at least five nights per week for the past 3–6 months, as confirmed by a sleeping partner; (ii) tooth wear and/or masseter muscle hypertrophy; (iii) a positive polygraphic diagnosis of sleep bruxism: at least two episodes of grinding noise per night, more than four episodes of sleep bruxism, and more than 25 bursts of bruxism per hour of sleep [10]. The validity of self-report, anamnestic and clinical tools to determine sleep bruxism was tested against data from electromyography in previous studies [30,31,32,33,34]. In 2008, Lavigne defined awake bruxism differently from sleep bruxism. Awake bruxism was defined as the state of awareness of jaw clenching in subjects in a waking state [10].

The Standardized Tool for Assessment of Bruxism (STAB) [13,14] represents an implementation of the guidelines established by the 2018 consensus. The STAB guidelines overlap with the recommendations made by Lavigne in 2008 [10]. According to STAB, the two main categories of tools used for diagnosing and assessing bruxism are axis A, which evaluate the status of bruxism and its consequences, and axis B, which include the etiological factors and comorbidities associated with bruxism [13]. For axis A, three categories of information are collected from subjects regarding bruxism: first, self-reported information (from questionnaires) (the subjective report corresponding to the anamnesis), second, clinical information as a result of a clinical examination (dentist’s report) (the objective report corresponding to the clinical assessment) and third, instrumental evaluation (technological report) (the paraclinical report corresponding to the paraclinical or complementary examinations) [13]. Axis B evaluation provides a self-report of psychological assessment of anxiety and depression, concurrent sleep-related conditions assessment, concurrent non-sleep conditions, report of taking medications and drugs, and additional factors [13].

The study aimed to validate the diagnostic system for sleep bruxism and awake bruxism proposed by STAB by correlating the results obtained from the questionnaire, the clinical exam and electromyographic examination. The null hypothesis was that there are no statistically significant differences between the results provided by non-instrumental evaluation (self-report and clinical exam) of sleep bruxism and awake bruxism and instrumental evaluation (sEMG) according to STAB.

## 2. Materials and Methods

### 2.1. Study Design and Participants

The study was conducted between October 2022 and June 2024 in the Dental Prosthetics and Oral Rehabilitation Clinic of the Faculty of Dental Medicine Craiova (FDM Craiova). The Ethics Committee of the University of Medicine and Pharmacy of Craiova approved the study according to report no. 156/25 July 2022. The study complied with the Declaration of Helsinki. Participation was voluntary. All participants were informed about the study and gave their consent.

The subjects included in the study were all students enrolled in the dentistry program from the FDM Craiova, IVth, Vth, and VIth years of study, respectively—305 students in total. The subjects had studied bruxism through the bruxism course delivered in the IVth year as part of the dental prosthetics course, and the bruxism management course in the oral rehabilitation program in the VIth year.

The inclusion criteria in the study were as follows: healthy subjects, without drug and orthodontic treatments, with a healthy dentition, without prosthetic restorations, with stable occlusion and with a maximum of one tooth edentulous gap, not wearing orthodontic appliances, residing in Craiova. The exclusion criteria were as follows: knowledge of the presence of bruxism, neurological and psychiatric disorders, drug treatments, extensive edentulism, unstable occlusion, presence of prosthetic restorations, orthodontic patients, and occlusal splints wear.

**Figure 1:**
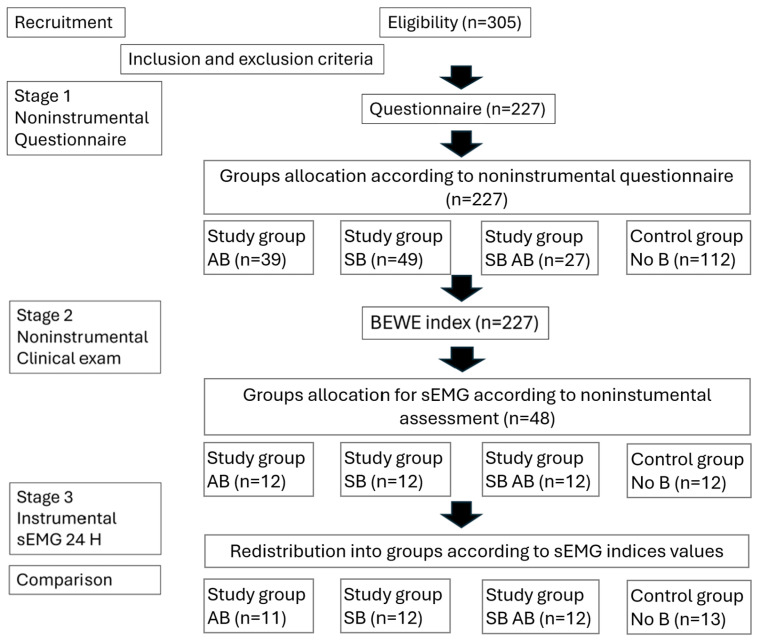
Outline of the steps for carrying out the assessment stages.

The study had three stages: 1. Questionnaire; 2. Clinical exam; 3. sEMG recording for 24 h (Figure 1).

The first stage of the study: after applying the inclusion/exclusion criteria, 227 students remained in the study and underwent a non-instrumental assessment based on a questionnaire. The sample size was computed using G*Power 3.1.9.7, Heinrich Heine University Düsseldorf, Germany, considering a significance level α of 0.05, a power 1 − β equal to 0.95, and a medium effect size value of 0.3 [35] (with an awareness of practical significance), resulting in a study requirement of a minimum of 220 participants.

The 227 students completed the questionnaire to establish a self-reported diagnosis of bruxism—possible bruxism. Following this stage, four groups were defined: participants with awake bruxism (AB), participants with sleep bruxism (SB), participants with combined bruxism (SB AB), and participants without bruxism (No B).

In the second stage of the study, the participants underwent a clinical examination of the dental arches, the buccal mucosa, and the tongue to highlight clinical signs of bruxism (teeth marks on the tongue, linea alba, and tooth wear). Among all participants, subjects who responded positively to the questionnaire and who presented clinical signs of bruxism were diagnosed with probable bruxism and qualified for the third stage of the study. The groups were adjusted according to the clinical findings.

The third stage of the study included an instrumental assessment of masseter muscle activity. sEMG recording over 24 h was performed for each participant in all four groups. A certain diagnosis of bruxism was established, and the four groups were redefined.

The diagnostic accuracy of the non-instrumental stage of the study was tested for sensitivity and sensibility using the sEMG results as a reference value.

### 2.2. Interview and Questionnaire

Data collection in the self-reporting stage was carried out based on a questionnaire that has proven helpful in other studies [36,37] and for didactic purposes within the learning program of FDM Craiova. The questionnaire included demographic data, data regarding possible bruxism diagnostic sounds heard by bed partner for sleep bruxism and bruxism acknowledgment in awake bruxism, data regarding the characteristics of sleep and awake bruxism (clenching and grinding activities), manifestations associated with bruxism at the level of the masticatory muscles, manifestations associated with temporomandibular dysfunction, the involvement of stress, anxiety, and other sleep disorders (insomnia, snoring, and restless legs syndrome), and smoking. For anxiety assessment, nine questions were formulated, with multiple answers depending on the severity, to which numerical values were associated for the answers, from 0 to 4 (never = 0, rarely = 1, sometimes = 2, often = 3, continuously = 4). Depending on the arithmetic mean obtained, anxiety was considered absent at an average below or equal to 2 and present at an average above 2 [38]. A single-item measurement with a 5-point scale was used for stress assessment, representing absent, moderate, and severe stress [39]. For sleep disorders assessment, a numerical scale was created depending on their presence, the maximum value being 9. Compared to STAB, data collected through the questionnaire obtained information regarding axis A1 (about sleep bruxism), A2 (about awake bruxism), A3 (about patient’s complaints), and B1 (psychological assessment by self-report) and B2 (concurrent sleep-related conditions assessment by self-report) [13].

### 2.3. Clinical Examination

During the clinical examination, lesions on the oral mucosa, such as the linea alba, teeth impressions, and oral lesions, were observed. A BEWE (basic erosive wear examination) index evaluation was performed to assess dental wear [40,41]. The BEWE index is a partial scoring system that records the most-affected tooth surface on a sextant and then sums the values obtained for the 6 sextants. The score used has 4 values: 0—no loss of tooth structure, 1—initial loss of surface texture, 2—distinct defect with loss of dental hard tissue less than 50% of the surface area, 3—loss of dental hard tissue more than 50% of the surface area. All surfaces (buccal, occlusal, lingual or palatal) are examined, and the highest score is recorded. For scores 2 and 3, dentin is usually also involved [40]. Compared to STAB, data collected through clinical examination provided information from axis A5 (intraoral examination) and A6 (tooth wear index) [13].

### 2.4. Electromyography

A portable surface electromyograph with a 24 h record was used for the instrumental evaluation (dia-Bruxo, Biotechnovation, San Remo, Italy). dia-Bruxo is a single-channel device (dimensions 43 × 50 × 10 mm) that records the electromyographic activity of the left masseter. The device uses disposable bipolar electrodes of AgAgCl (interelectrode distance of 22 mm) provided with an adhesive gel. The recorded signals are transmitted to an analog circuit that processes the signal, amplifies it, filters it between 110 and 550 Hz, and adapts it so that it is transmitted in a digital format with a discrimination level of 4096 (12-bit analog/digital converter) and acquisition every 100 mS. The data are subsequently entered into computer software that transforms the signal into RMS (root mean square) waves. For each subject, the software is programmed with their data and the exact period of sleep and wakefulness for the correct interpretation of the results. More details about this device can be found in a previously published article by our team [42]. The dia-Bruxo software analyzes bruxism episodes, differentiating between clenching, grinding, and other electromyographic activities specific to bruxism [37,42,43]. The dentist who performed the EMG recordings did not know that the bruxism diagnostic was already established (blinded). After the left masseter area was prepared through skin disinfection with alcohol, the sensor was fixed on the preauricular area. The device was calibrated as indicated by the producer [37]. The cut-off criteria for electromyographic activity were the number of bruxism events of clenching and grinding per hour of sleep for sleep bruxism and the sEMG indices values published by the producer onsite [42,44,45]. Sleep bruxism presence was considered when the device recorded two events of clenching, grinding, or other bruxism activity per hour of sleep [42,45]. Awake bruxism was also considered according to the sEMG indices values recommended by the producer [37,42].

The sEMG indices computed by the device were the bruxism indices, personal (bruxism personal index or BPI), time (bruxism time index or BTI), and work (bruxism work index or BWI). The device also computes masseter muscle activity (MMA), expressed as a masseter personal index (MPI), masseter time index (MTI), masseter work index (MWI), and muscular power (MP). Compared to STAB, data collected through electromyography provided information from axis A7.1 (electromyography for sleep bruxism) and A8.2 (electromyography for awake bruxism) [13].

### 2.5. Statistical Analysis

Statistical analysis included all subjects who completed the questionnaire to determine the association of bruxism with muscle and temporomandibular problems, orofacial pain, stress, anxiety, sleep disorders, smoking, and tooth wear. Possible and probable diagnostics of bruxism were developed. In the subgroup with sEMG analysis, the diagnosis of sleep bruxism established with certainty was compared with possible and probable diagnosis of sleep bruxism.

The analysis included data from questionnaires, clinical charts, and sEMG recordings. Data were collected in Microsoft Excel (Microsoft Corporation, Redmond, Washington, DC, USA). SPSS (Statistical Package for Social Sciences) software, version 26 (SPSS Inc., Armonk, NY, USA) was used to complete the corresponding data analysis. Continuous variables were defined as median values and “mean ± standard deviation” (SD). Ordinal and nominal parameters were determined as frequency distributions and corresponding percentages. For all sEMG parameters representing continuous data series, normality was assessed using the Kolmogorov–Smirnov/Shapiro–Wilk test. The chi-square test was used to evaluate associations between groups. Comparisons between groups were performed using the Mann–Whitney U or the Kruskal–Wallis H tests. The diagnosis of AB, SB, and combined bruxism determined based on the questionnaire was compared with the diagnosis provided by the sEMG recordings. For each type of bruxism, this comparison yielded four different values: true positive (TP), when the type of bruxism was correctly determined through the questionnaire; false positive (FP), false negative (FN), or true negative (TN), when the questionnaire indicated a specific type of bruxism, or no bruxism, when not confirmed by the sEMG recording. Then, the sensitivity, specificity, positive predictive value (PPV), and negative predictive value (NPV) were computed as follows: sensitivity represents the ratio between TP and TP + TN; specificity represents the ratio between TN and TN + FP; PPV represents the ratio between TP and TP + FP; and NPV represents the ratio between TN and TN + FN.

For the chi-square test, the effect size, or magnitude of the association between the two variables, was given by the index ω, which was computed as the square root of the chi-square value divided by the sample size. For the Mann–Whitney yest, the effect size was given by *r* and was computed by dividing the absolute value of the standardized test statistic z by the square root of the number of observations (the sample size). For the Kruskal–Wallis yest, the effect size was given by *η*^2^ and was computed as the ratio between the value H, representing the test statistic, minus the number of groups plus 1 (H − k + 1), and the difference between the total number of observations and the number of groups (n–k). The effect size was considered very small for values < 0.1, small for values between 0.1 and 0.3, moderate for values between 0.3 and 0.5, and large for values > 0.5, based on thresholds suggested by Cohen [35]. A *p*-value < 0.05 was interpreted as statistically significant in a confidence interval (CI) of 95%.

## 3. Results

The first- and second-stage study group included 227 participants aged 20–45 who completed a questionnaire. From analysis of the demographic data, the study included 227 subjects, of whom 158 (69.6%) were females and 69 (30.4%) were males. The provided answers and the self-evaluation of the frequency of bruxism episodes revealed that almost half of the participants did not have bruxism (Figure 1).

Participants were divided as follows: AB subgroup (39 participants, representing 17.18% of the entire study group), SB subgroup (49 participants, 21.59%), AB and SB subgroup (27 participants, 11.89%), and a subgroup without bruxism (112 participants, 49.34%) (Table 1).

The distribution of subjects by type of bruxism shows that of the 115 subjects with bruxism, most had sleep bruxism, followed by those with awake bruxism and those with both types of bruxism. Thus, over half of the study participants had possible bruxism (50.66%). This distribution observed for the whole group was observed also in the case of female subjects. Male subjects had bruxism in lower percentages, with sleep bruxism at 15.94%, then combined bruxism, followed by awake bruxism (Table 1).

The enrolled participants belonged mainly to the young age group (20–24 years old), (59.91%), the rest being adults up to 45 years old (40.09%).

Similar percentages of young participants were identified within each subgroup. Gender and age group distributions were similar, without statistically significant differences between the four subgroups (*p* > 0.05, Table 1).

According to the data from the questionnaire, the participants did not recognize the presence of bruxism except partially. Frequent episodes of bruxism (defined as “Sometimes”) were reported by participants with possible SB and combined bruxism (around one third each), while only 22.86% of participants with possible AB acknowledged frequent episodes. Participants with possible AB were rarely or never aware of their bruxism episodes, participants with possible SB were more aware of their bruxism, reporting frequent episodes, and participants with possible combined bruxism reported frequent and very frequent episodes. There was a statistically significant difference between the four subgroups and the self-reported frequency of bruxism episodes, χ^2^(3) = 96.015, *p* < 0.0005 (Table 1).

Regarding motor muscle activities (clenching and grinding) during sleep or wakefulness, over half of the participants diagnosed with possible bruxism following the questionnaire analysis did not recognize the presence of these motor activities. Almost all subjects without bruxism recognized the absence of clenching and grinding activity in 95–100% of cases (Table 2).

Regarding axis A3 of the STAB, the questionnaire addressed questions about pain, tension, and muscle fatigue present upon awakening and during the day. Thus, according to the data, the participants answered as follows: those with SB complained of pain in the masticatory muscles upon awakening in a proportion of 34.69%, of muscle fatigue upon awakening in a proportion of 42.86% and of headaches upon awakening in a proportion of 30.61%, and those with AB complained of muscle tension during the day in a proportion of 5.13% and of muscle fatigue during the day in a proportion of 12.82% (Table 3).

Subjects with combined bruxism most often reported muscle fatigue upon awakening, muscle tension during the day, followed by muscle fatigue during the day and headache upon awakening, and then muscle pain upon awakening, all in various percentages as shown in Table 3. Subjects without bruxism reported 100% or almost 100% of having no pain, tension, or muscle fatigue either upon awakening or during the day. Regarding headache upon awakening, 8.93% presented with this type of pain.

Following the answers from the questionnaire, less than 10% of the participants from the entire study group were anxious (17 participants, 7.49%) (Table 4).

Around a quarter of all participants experienced severe stress (62 participants), 43.61% reported a moderate stress level, while 27.31% reported no stress (Table 4). Most participants with moderate or no stress did not have possible bruxism (more than 50%). More participants with possible AB reported moderate stress compared to the other participants with possible bruxism. Overall, participants without bruxism reported feeling less stressed compared to participants with possible bruxism. Only 7.41% of participants with possible combined bruxism reported no stress, significantly less than participants with possible AB or possible SB. Thus, there were statistically significant differences between the different forms of bruxism regarding the stress level, χ^2^(3) = 22.592, *p* < 0.0005 (Table 4).

Only 30 participants (13.22% from the entire study group) were dissatisfied with their current occupation, and most of them were participants with possible SB (36.67% from participants with no satisfaction related to their occupation), or without bruxism (26.67%). Participants with possible combined bruxism represented only 13.33% of all dissatisfied participants. Thus, there was a statistically significant association between possible bruxism subgroup and job dissatisfaction, χ^2^(3) = 8.066, *p* = 0.045. The association was small [35] with Cramer’s V = 0.188 (Table 4).

Less than half of the entire study group had experienced oro-facial pain in the past month (Table 5). As the frequency increased, more participants with that reported frequency had possible bruxism, compared to those without bruxism. Overall, there was a statistically significant difference between participants with and without bruxism, U = 4754, z = −4.002, *p* < 0.0005. Similar trends were observed for chronic oro-facial pain in the past 6 months, as only participants with possible bruxism experienced chronic pain sometimes and often, as well as most of the participants with rare episodes. Thus, there was a statistically significant difference between participants with and without bruxism related to chronic oro-facial pain, U = 5143, z = −3.878, *p* < 0.0005 (Table 5).

A Mann–Whitney U test was run to determine if there were differences in the sleep disorders score between participants with and without possible bruxism. The distributions of the scores for both groups were similar, as assessed by visual inspection. The median sleep disorder score was statistically significantly higher in participants with possible bruxism (4.00) than in participants without bruxism (2.00), U = 4082.50, z = −4.812, *p* < 0.0005. A similar test was run to determine the difference between the same groups regarding the insomnia score. With a median insomnia score of 3.00 for both participants with and without possible bruxism, there was no statistically significant difference between the groups, U = 7209.00, z = 1.572, *p* = 0.116 (Table 5).

The analysis of restless feet syndrome revealed that 70.27% of participants with possible bruxism experienced this syndrome, compared to only 29.73% of participants without possible bruxism. Thus, there was a statistically significant association between the presence of possible bruxism and restless feet syndrome, χ^2^(1) = 6.801, *p* = 0.009. The association was small [35], Cramer’s V = 0.173. Smoking, however, did not seem to influence the existence of possible bruxism, as there were similar percentages of participants with and without possible bruxism who smoked, χ^2^(1) = 0.597, *p* = 0.440, Cramer’s V = 0.051 (Table 5).

A Kruskal–Wallis test was conducted to determine if there were differences in BEWE scores between groups with different types of possible bruxism and no bruxism (both by questionnaire and by sEMG). The distributions of BEWE scores were similar for all groups, as assessed by visual inspection of a boxplot. Median BEWE scores were statistically significantly different between the different forms of possible bruxism defined by questionnaire, χ^2^(3) = 114.029, *p* < 0.0005. Subsequently, pairwise comparisons were performed using Dunn’s procedure. A Bonferroni correction for multiple comparisons was performed with statistical significance accepted at the *p* < 0.0083 level. This post hoc analysis revealed statistically significant differences in BEWE score between the group with no bruxism and all the other groups, as well as between possible combined bruxism and AB, as well as SB. Median BEWE scores were not statistically significantly different between the different forms of possible bruxism defined by sEMG, *p* > 0.05.

The risk of tooth wear interpreted as low according to Bartlett scale is justified by the mean age of 25 years of the participants in the study (Table 6 with values corresponding to all 227 participants; Table 7 with values corresponding to the 48 participants from the third stage).

In Table 8, the results of the sEMG reports are presented in two ways: in the non-instrumental allocation groups (after self-report and clinical exam) and in the instrumental allocation groups (after sEMG). Almost all the indices are different between the two analyses, the second reorganization of groups being more accurate for bruxism diagnosis. As a cut-off criterion for sleep bruxism, the number of bruxism events per hour was characteristic: AB and no bruxism groups had a value under 2, while the SB group and the SB AB group had over 4 bruxism events per hour—respectively, 5.4 and 4.58. These events were more related to clenching activity in sleep, with SB and SB AB groups having a significantly higher number of clenching episodes during sleep compared with the AB and no bruxism groups. The bruxism indices (personal, time and work bruxism indices) were different according to the type of bruxism and the period of the day when registration was performed. For sleep bruxism, the work index was significantly higher in the SB and SB AB groups compared to the AB and no B groups. The cut-off criterion for awake bruxism was the work index for awake bruxism as given by the device software and recommended by the producer.

**Figure 2:**
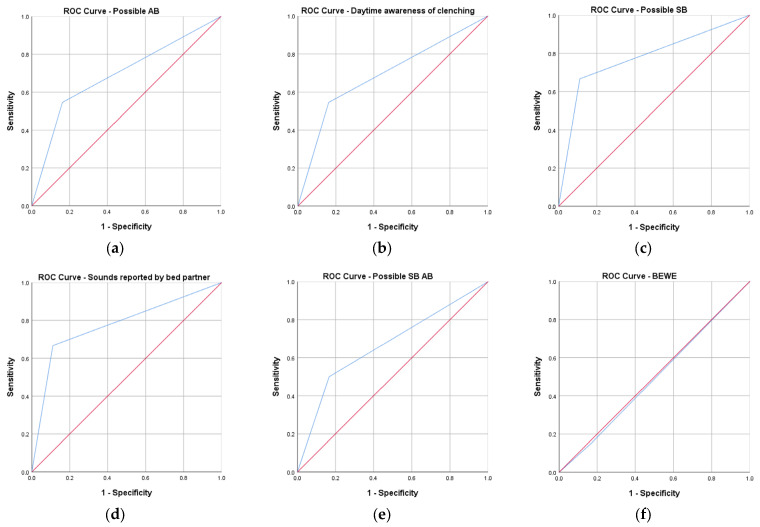
ROC curves for: (**a**) Possible AB; (**b**) Daytime awareness of clenching; (**c**) Possible SB; (**d**) Sounds reported by bed partner; (**e**) Possible SB AB; (**f**) BEWE risk; (blue line—ROC curve, red line—diagonal produced by ties).

According to Table 9, the highest sensitivity observed corresponds to the clenching and grinding sounds reported by the bed partner (a sign for possible SB). The same parameter also recorded the highest specificity, followed by daytime awareness of clenching (a sign of possible AB). The frequency of bruxism episodes used to define the diagnosis of possible SB and AB had a specificity of 83%. The sign expressed by the sounds reported by the bed partner had the highest PPV value, while the signs of daytime awareness of clenching or combined bruxism recorded values of 50%. Figure 2 presents the associated ROC curves.

The presence of bruxism sounds during the night and awareness of clenching during the day may be used to screen patients with AB and SB. A low frequency of reported episodes of bruxism or the lack of muscular pain or fatigue could indicate the absence of bruxism.

## 4. Discussion

This clinical study tested the non-instrumental assessment in bruxism performed according to the STAB criteria [13] with tools (questionnaire, clinical examination) validated by previous studies [19,36,46], against the standard instrumental assessment in bruxism, namely, portable electromyography [37,42], also present within STAB. To our knowledge, this is the first study with this type of design, using a portable sEMG device with 24 h recording that included both awake and sleep manifestations of bruxism. Previously, studies have been published in which the diagnostic capacity of questionnaires and clinical examination was evaluated against the gold standard in sleep bruxism, namely, polysomnography [34,44,47], but also electromyography [30,31,32,33,34]. The results of our study showed that the sensitivity and specificity of the tools were the highest for sleep bruxism. Thus, our non-instrumental questionnaire assessment identified 67% of sleep bruxism cases and 89% of cases that did not have sleep bruxism. In the case of awake bruxism, the specificity was higher, while the sensitivity of the test was lower because almost half of the subjects were not aware of the presence of awake bruxism. Regarding tooth wear, the tests showed low sensitivity but high specificity. It is evident that the absence of tooth wear is frequently associated with the absence of bruxism, while the presence of tooth wear does not imply the existence of bruxism. The multifactorial etiology of tooth wear is the reason for this result. In the last two decades, dental erosion has been much more frequent because of changes in eating habits, oral hygiene, and living and working conditions [48,49]. In a study on SB clinical exam and diagnostic criteria validation with polysomnography, Palinkas et al. [34] obtained similar results for signs and symptoms of SB as in our study.

Bruxism diagnosis is performed using several tools, such as questionnaires or interviews, clinical examination, and electromyography or polysomnography [1,9,13,14]. Since their diagnostic values are variable, the resultant diagnosis of bruxism is graded as possible (after questionnaires or interviews), probable (after clinical examination), and certain (after electromyography and polysomnography) [1,9]. In epidemiology, bruxism is determined in large populations by questionnaires/self-reporting or clinical examination (e.g., dental wear) [19,29].

Bruxism frequency is variable, depending on the time frame, population, and other factors like the evaluation method and study type [27,28]. In our study, bruxism frequency was 50.66% for possible bruxism, according to non-instrumental data. The frequency for SB was 21.59%, for AB 17.18%, and for SB AB 11.89%. One study reported an average value for the prevalence of SB of 21%, similar to our study [29]. In Romania, the average prevalence of SB was 16.28%, and AB was 68.99%, while 14.73% of people presented a combined form of bruxism [19]. It should be noted that the study conducted by questionnaire on dental students suggests a high value because the subjects knew about the condition and were familiar with data about it from the faculty, and thus were more able to correctly evaluate by self-assessment the presence or absence of bruxism.

As Lavigne stated in 2008, bruxism occurs predominantly in women. In our study, women presented bruxism in a higher percentage than men [10]. As mentioned in another article [42], this distribution reflects the proportion of people enrolled in dentistry: two-thirds females and one-third males. In this study, females present bruxism in a much higher percentage (89 people, 56.33%) than males (26 people, 37.68%).

SB is frequently concurrent with AB (in one-third of subjects), according to Carlsson et al. [50]. According to these authors, AB is characterized by clenching of the teeth. Our results showed a higher number of clenching events in awake time compared to sleep time. AB tends to occur more frequently with advancing age, being found in only 12% of children [51] compared to 20% of adults [52]. Also, in our study, the frequency of SB was higher than that of AB in young groups of participants compared to adult participants, where both frequencies are almost equal. It is interesting to note that in our study, there were no differences in the presence of bruxism between young and adult groups, with both groups having bruxism in a proportion of over 50%. In young people, sleep bruxism is the most frequent, followed in descending order by awake bruxism and then by combined bruxism. In adults, sleep and awake bruxism have similar percentages, followed by combined bruxism, which is half as common as sleep or awake bruxism. The descending trend of sleep bruxism frequency confirms that, as people age, awake bruxism is more common, and sleep bruxism, which is typical of children and young people, decreases.

As a subject of interest for several medical specialties, sleep bruxism has been studied more and is considered a sleep disorder [53,54]. ASDA (American Sleep Disorders Association) defined sleep bruxism as a stereotyped movement disorder characterized by teeth grinding or clenching during sleep [44,55]. The diagnosis relies on reporting grinding or clenching sounds during sleep in combination with one of the following signs: abnormal tooth wear, hypertrophy of the masseter muscles on voluntary clenching, discomfort, and fatigue or pain of the masticatory muscles [17,56].

In 2005, the American Academy of Sleep Medicine (AASM), together with the European Society for Sleep Research, the Japanese Society for Sleep Research, and the Latin Society for Sleep, published a second edition of the International Classification of Sleep Disorders, which included the minimum criteria for the clinical exam in diagnosis of sleep bruxism [56]. These criteria are the following: 1. the presence of grinding and clenching of the teeth during sleep, and 2. the existence of at least two of the following signs: pathological wear of the teeth, noises associated with grinding the teeth, discomfort of neck muscles [46,56]. In 2013, the first international consensus on bruxism attempted to standardize the approach to bruxism by creating a standard definition for all specialists, a graded diagnostic system, and the acceptance of awake bruxism as a separate entity from sleep bruxism [9]. The 2018 consensus continued the first international consensus on bruxism [1]. Based on the evidence in the bruxism literature, the second consensus established two separate definitions for sleep bruxism and awake bruxism. In 2010 and 2012, our team performed and published several studies on bruxism that differentiated sleep bruxism from awake bruxism [36,46].

In his 1996 classic study, Lavigne tested the validity of the clinical diagnostic criteria of sleep bruxism based on polysomnographic recordings [44]. Clinical examination revealed dental wear in almost all (16 of 18 bruxists) and masticatory muscle discomfort in less than half of the group. Polysomnographic examination showed that asymptomatic subjects had an average of 1.7 bruxism episodes per hour of sleep, while bruxists had 5.4 bruxism episodes per hour of sleep. In our study, the median values of sleep bruxism events per hour were the same as in Lavigne’s study (1.74 episodes per hour for the no bruxism group and 5.4 episodes per hour in the SB group).

The diagnosis of SB is usually clinical, although the gold standard remains a whole night of PSG recording with audio-video recording [57]. In a pilot study, Yoshizawa et al. [47] studied the association between the clinical diagnosis criteria for sleep bruxism and video-polysomnographic bruxism activity. The report of grinding sounds certified by an eyewitness correlated positively with a higher frequency of RMMA (rhythmic masticatory muscle activity) episodes, as did the presence of teeth attrition. In contrast, Raphael et al. showed that self-report of teeth grinding awareness in sleep bruxism, compared to polysomnographic registration, was a poor indicator of sleep bruxism [32].

In a recent clinical study, Ohlmann et al. evaluated the validity of self-report and clinical signs of sleep bruxism against electromyographic/electrocardiographic data registered with a portable device, Bruxoff [30]. Analyzing results for a German dental clinical population, this study showed that self-report questionnaires (based on questions in Axis II of the Research Diagnostic Criteria for Temporomandibular Disorders) had reduced sensitivity, higher specificity, and moderate accuracy compared to electromyographic devices. Conversely, clinical signs correlated better with electromyographic data, showing better sensitivity, specificity, and accuracy (over 70%). Their results are in contradiction to results obtained by Castroflorio et al. in 2015, which showed that clinical diagnostic of sleep bruxism was not correlated with the electromyographic diagnosis of sleep bruxism obtained with Bruxoff [31].

The future direction for the assessment of bruxism should be the development of a portable tool that directly, quickly, and accurately measures bruxism activity and that can be used both in the clinic (for diagnosis, evaluation of treatment results, and follow-up) and in research. Other authors, like Bracci [58], are more skeptical, considering that a graded diagnostic system of bruxism for possible, probable, and definite awake and sleep bruxism cannot be achieved under the current conditions of knowledge and available strategies.

In a clinical study on a group of dental students in Norway, Jonsgar et al. [33] sought to compare the electromyographic activity in subjects with attrition and subjects without attrition. The EMG investigation used a portable, single EMG device, Grindcare, that recorded the EMG activity of the anterior temporal muscle, unilaterally. According to their study, there was not a notable difference in EMG activity of the temporal muscle between attrition-positive subjects and attrition-negative subjects. However, the prevalence of sleep bruxism self-reported activity was more common in the attrition group compared to the non-attrition group. Also, in this study as in our study, the researchers concluded that tooth wear (in their case attrition) should not be used as a direct indication of active sleep bruxism.

According to STAB [13,57], the diagnostic approach to bruxism includes non-instrumental and instrumental measures. Non-instrumental measures include self-report (questionnaires and anamnesis) and clinical examination. These methods can be used for both awake and sleep bruxism. It should be noted that the clinical examination mainly assesses the consequences of bruxism and bruxism per se almost not at all [13]. It is challenging to differentiate the clinical aspects of awake bruxism from those of sleep bruxism, except for intrinsic mechanical wear of the attrition type, which is more difficult to assess in the waking state because grinding occurs rarely during the waking state. The same considerations can be highlighted regarding the complications of prosthetic work. In our study, the most intense motor activity was clenching in both types of bruxism. The number of clenching events, as well as grinding, was higher in the SB AB and in the AB groups in daytime but also in the SB group, compared to the no bruxism group. Self-reporting through structured questionnaires and interviews can be useful for obtaining information on bruxism activities and associated factors, but the intensity and duration of specific actions cannot be quantified.

Another problem is generated by the fact that the psyche influences self-reporting, which is very subjective [20,59,60]. For this reason, the assessment of bruxism through self-reporting has limited value. However, self-reporting is the best strategy for obtaining data for epidemiological studies and for identifying bruxism at the individual level. There are no specific questionnaires for AB. The most used approach involves the use of instruments designed for a broader purpose, such as for reporting bruxism, e.g., Bruxscale, for temporomandibular disorders, the DC/TMD (Diagnostic Criteria for Temporomandibular Disorders), or the Oral Behavior Checklist, for oral behaviors. As a possible means of improving the data collected, patients can be taught to monitor their oral behavior of clenching, as well as other oral motor behaviors like bracing, thrusting, and tooth-to-tooth contacts for 1–2 weeks. This ecological momentary approach (EMA) or experience-based identification methodology (ESM) can improve the quality and quantity of data collected through data obtained from a diary or phone application. The problem with this approach is that the patient needs more time to become aware of these manifestations and requires repeated reinforcement. Most of our patients (90% from the clinical ward, unpublished data) do not recognize the existence of bruxism, regardless of its type, sleep or awake. Only after the appearance of dental or prosthetic complications and detailed discussions about this problem do they understand the condition and become aware of it, thus being able to pay attention to the clinical manifestations and note them in a diary or application.

It is worth observing that, in our group of subjects, stress was strongly correlated with bruxism, compared to anxiety. According to STAB, stress related to bruxism is no longer taken into consideration [13]. In our opinion, this situation represents a weak point of STAB, because many studies have highlighted the association between stress and bruxism compared to the anxiety–bruxism association [19,61,62], which is not as consistent (in our study, the association was insignificant, and the same is found in other studies) [63]. We suggest that stress should be introduced as an aspect of evaluation for subjects suspected of experiencing bruxism.

The clinical evaluation should include an extraoral and an intraoral examination to identify signs and symptoms related to bruxism [13,57]. The extraoral examination should evaluate the masticatory muscles (masseter hypertrophy, for example), the presence of pain (muscle pain, TMJ pain, headaches), and other signs of TMD (temporomandibular dysfunction) [1,59]. The intraoral examination should include a detailed dental-periodontal examination to monitor the dental effects of bruxism (dental wear, enamel cracks and fractures, broken tooth syndrome, vertical or horizontal root fractures in vital and non-vital teeth, failure of prosthetic restorations, tooth mobility—occlusal trauma) and inspection of the mucous membranes of the cheeks to identify the linea alba, dental impressions on the tongue and traumatic lesions, e.g., of the tongue and cheeks, as well as the presence of intraoral pain (teeth soreness or hypersensitivity) [1,57]. The intraoral examination usually detects the consequences of bruxism at the dental, mucosal and periodontal levels and cannot identify bruxism per se nor the difference between awake and sleep bruxism. Even though the two forms of bruxism may have different etiology, they can coexist, and sleep bruxism from the youth period is replaced by awake bruxism towards the old age period, because of changes in the neurological and psychological traits and behaviors related to stressful events and stress coping. In the present study, bruxism was associated with stress, but not with anxiety [20].

To identify bruxism and its type, we should consider a differential diagnosis scheme/procedure. Instrumental evaluation, which may be available and has been used for many years to record bruxism activities, can be recommended for the evaluation of both SB and AB. Because polysomnography is difficult to perform for sleep bruxism, polysomnography laboratories are rare and not available to practitioners, and for awake bruxism, electromyography (EMG) evaluation is superficial because it cannot capture the characteristics of bruxism at the time of their occurrence, these approaches to evaluating bruxism have limitations, though are widely used in research [64]. Therefore, researchers have tried to use devices for electromyographic recording of the activity of the masseter or temporal muscles over a longer period (e.g., 24 h) that can be easily worn by the patient to monitor muscle contractions [58]. This type of device includes the dia-Bruxo, used in this study, that can evaluate awake and sleep bruxism activity and provide a report with multiple indices of bruxism [37,42], some of them recommended by STAB [13].

The current study used STAB criteria for validation of non-instrumental tools of bruxism assessment (a questionnaire and clinical exam) with the instrumental tool (portable sEMG 24 h recording) and the results obtained proved to be valuable in the research context. The study used STAB to explore bruxism association with the main bruxism signs (clenching and grinding), but also with temporo-mandibular dysfunction and masticatory muscles motor activity and with psychological traits of the subjects included in the study.

According to the results of the present study, the null hypothesis was not confirmed because the results obtained through self-report and clinical exam revealed an overestimation of awake bruxism.

Limitations of the study consist of the reduced number of participants in the third stage of the study, as well as the fact that our questionnaire, constructed according to older recommendations, is not perfectly aligned with the current STAB questions. Another limitation could be the short period of instrumental registration, only 24 h, a day and a night, compared to other studies that registered over a longer period of time.

## 5. Conclusions

Non-instrumental evaluation in our study provided data regarding the frequency of bruxism, according to gender and age of the subjects but also by bruxism type. In clinical settings, non-instrumental evaluation of bruxism through questionnaire and clinical exam has its value, especially for sleep bruxism, indicating that the possible and probable diagnostic of bruxism is a starting point for bruxism management. For an accurate and certain diagnosis of bruxism, instrumental evaluation as a gold standard can complete the clinical evaluation. The STAB criteria proved to be useful in bruxism diagnosis, but they should be tested on larger groups. Portable devices are useful both in research and clinical settings, and their properties should be validated through extensive research.

## Figures and Tables

**Table 1:** Demographic aspects of the subjects included in the study.

Parameter	Value	AB	SB	AB and SB	No Bruxism	Total	*p*
		39 (17.18%)	49 (21.59%)	27 (11.89%)	112 (49.34%)	227 (100%)	
Gender	F	32 (20.25%)	38 (24.05%)	19 (12.03%)	69 (43.67%)	158 (100%)	0.052 * ω = 0.184
		82.05%	77.55%	70.37%	61.61%		
	M	7 (10.14%)	11 (15.94%)	8 (11.59%)	43 (62.32%)	69 (100%)	
		17.95%	22.45%	29.63%	38.39%		
Age group	Young	21 (15.44%)	31 (22.79%)	17 (12.5%)	67 (49.26%)	136 (100%)	0.818 * ω = 0.064
		53.85%	63.27%	62.96%	59.82%		
	Adult	18 (19.78%)	18 (19.78%)	10 (10.99%)	45 (49.45%)	91 (100%)	
		46.15%	36.73%	37.04%	40.18%		
Self-assessment of bruxism episodes	Never	12 (12.77%)	8 (8.51%)	0 (0%)	74 (78.72%)	94 (100%)	<0.0005 ** η^2^ = 0.417
		30.77%	16.33%	0%	66.07%		
	Rarely	18 (26.47%)	10 (14.71%)	6 (8.82%)	34 (50%)	68 (100%)	
		46.15%	20.41%	22.22%	30.36%		
	Sometimes	8 (22.86%)	13 (37.14%)	12 (34.29%)	2 (5.71%)	35 (100%)	
		20.51%	26.53%	44.44%	1.79%		
	Often	1 (4.76%)	14 (66.67%)	4 (19.05%)	2 (9.52%)	21 (100%)	
		2.56%	28.57%	14.81%	1.79%		
	Continuously	0 (0%)	4 (44.44%)	5 (55.56%)	0 (0%)	9 (100%)	
		0%	8.16%	18.52%	0%		

* Chi-Square test. ** Kruskal–Wallis H test. F = female, M = male, AB = awake bruxism, SB = sleep bruxism. Values in grey are percentages summed by columns for each parameter.

**Table 2:** Bruxism masticatory muscle activity of clenching and grinding reported by the study group.

Parameter	Value	AB	SB	AB and SB	No Bruxism	Total	*p* *
		39 (17.18%)	49 (21.59%)	27 (11.89%)	112 (49.34%)		
A1 SB Clenching	Yes	2 (5.26%)	18 (47.37%)	15 (39.47%)	3 (7.89%)	38 (100%)	<0.0005 ω = 0.526
		5.13%	36.73%	55.56%	2.68%		
	No	37 (19.58%)	31 (16.4%)	12 (6.35%)	109 (57.67%)	189 (100%)	
		94.87%	63.27%	44.44%	97.32%		
A1 SB Grinding	Yes	0 (0%)	11 (47.83%)	11 (47.83%)	1 (4.35%)	23 (100%)	<0.0005 ω = 0.707
		0%	22.45%	40.74%	0.89%		
	No	39 (19.12%)	38 (18.63%)	16 (7.84%)	111 (54.41%)	204 (100%)	
		100%	77.55%	59.26%	99.11%		
A2 AB Clenching	Yes	20 (31.25%)	20 (31.25%)	19 (29.69%)	5 (7.81%)	64 (100%)	<0.0005 ω = 0.551
		51.28%	40.82%	70.37%	4.46%		
	No	19 (11.66%)	29 (17.79%)	8 (4.91%)	107 (65.64%)	163 (100%)	
		48.72%	59.18%	29.63%	95.54%		
A2 AB Grinding	Yes	9 (34.62%)	5 (19.23%)	12 (46.15%)	0 (0%)	26 (100%)	<0.0005 ω = 0.463
		23.08%	10.2%	44.44%	0%		
	No	30 (14.93%)	44 (21.89%)	15 (7.46%)	112 (55.72%)	201 (100%)	
		76.92%	89.8%	55.56%	100%		

* Chi-Square test. A1 and A2 are the non-instrumental bruxism axes established in STAB. Data from the study were interpreted according to them. Values emphasized in dark grey represent a motor activity characteristic for each type of bruxism, while those in light grey represent an activity common for combined bruxism. AB = awake bruxism, SB = sleep bruxism.

**Table 3:** Bruxism masticatory muscle pain, tension and fatigue and headache in the study group.

Parameter	Value	AB	SB	AB and SB	No Bruxism	Total	*p* *
		39 (17.18%)	49 (21.59%)	27 (11.89%)	112 (49.34%)		
A3 (TMD) Muscle pain waking up	Yes	0 (0%)	17 (60.71%)	11 (39.29%)	0 (0%)	28 (100%)	<0.0005 ω = 0.531
		0%	34.69%	40.74%	0%		
	No	39 (19.6%)	32 (16.08%)	16 (8.04%)	112 (56.28%)	199 (100%)	
		100%	65.31%	59.26%	100%		
A3 (TMD) Muscle fatigue waking up	Yes	0 (0%)	21 (55.26%)	17 (44.74%)	0 (0%)	38 (100%)	<0.0005 ω = 0.649
		0%	42.86%	62.96%	0%		
	No	39 (20.63%)	28 (14.81%)	10 (5.29%)	112 (59.26%)	189 (100%)	
		100%	57.14%	37.04%	100%		
A3 (TMD) Muscle tension during the day	Yes	2 (5.26%)	21 (55.26%)	13 (34.21%)	2 (5.26%)	38 (100%)	<0.0005 ω = 0.534
		5.13%	42.86%	48.15%	1.79%		
	No	37 (19.58%)	28 (14.81%)	14 (7.41%)	110 (58.2%)	189 (100%)	
		94.87%	57.14%	51.85%	98.21%		
A3 (TMD) Muscle fatigue during the day	Yes	5 (15.15%)	15 (45.45%)	12 (36.36%)	1 (3.03%)	33 (100%)	<0.0005 ω = 0.453
		12.82%	30.61%	44.44%	0.89%		
	No	34 (17.53%)	34 (17.53%)	15 (7.73%)	111 (57.22%)	194 (100%)	
		87.18%	69.39%	55.56%	99.11%		
A3 (TMD) Headache waking up	Yes	0 (0%)	15 (40.54%)	12 (32.43%)	10 (27.03%)	37 (100%)	<0.0005 ω = 0.393
		0%	30.61%	44.44%	8.93%		
	No	39 (20.53%)	34 (17.89%)	15 (7.89%)	102 (53.68%)	190 (100%)	
		100%	69.39%	55.56%	91.07%		

* Chi-Square test. A3 is a bruxism axis established in STAB. Data from the study were interpreted according to it. Values emphasized in dark grey represent muscle pain, tension and fatigue characteristic for each type of bruxism, while values in light grey are common for combined bruxism. AB = awake bruxism, SB = sleep bruxism, TMD = temporo-mandibular disorder.

**Table 4:** Stress level, anxiety level and job dissatisfaction in study group (axis B in STAB, possible risk factors for bruxism).

Parameter	Value	AB	SB	AB and SB	No Bruxism	Total	*p*
		39 (17.18%)	49 (21.59%)	27 (11.89%)	112 (49.34%)		
Stress	No stress	9 (13.64%)	14 (21.21%)	2 (3.03%)	41 (62.12%)	66 (100%)	<0.0005 ** η^2^ = 0.088
		23.08%	28.57%	7.41%	36.61%		
	Moderate	20 (20.2%)	16 (16.16%)	9 (9.09%)	54 (54.55%)	99 (100%)	
		51.28%	32.65%	33.33%	48.21%		
	Severe	10 (16.13%)	19 (30.65%)	16 (25.81%)	17 (27.42%)	62 (100%)	
		25.64%	38.78%	59.26%	15.18%		
B1 Anxiety	Present	2 (11.76%)	4 (23.53%)	4 (23.53%)	7 (41.18%)	17 (100%)	0.443 * ω = 0.109
		5.13%	8.16%	14.81%	6.25%		
	Absent	37 (17.62%)	45 (21.43%)	23 (10.95%)	105 (50%)	210 (100%)	
		94.87%	91.84%	85.19%	93.75%		
B1 Anxiety level	Never	0 (0%)	2 (50%)	1 (25%)	1 (25%)	4 (100%)	0.371 ** η^2^ = 0.001
		0%	4.08%	3.7%	0.89%		
	Rarely	23 (23%)	21 (21%)	11 (11%)	45 (45%)	100 (100%)	
		58.97%	42.86%	40.74%	40.18%		
	Sometimes	14 (13.21%)	22 (20.75%)	11 (10.38%)	59 (55.66%)	106 (100%)	
		35.9%	44.9%	40.74%	52.68%		
	Often	2 (12.5%)	4 (25%)	4 (25%)	6 (37.5%)	16 (100%)	
		5.13%	8.16%	14.81%	5.36%		
	Continuously	0 (0%)	0 (0%)	0 (0%)	1 (100%)	1 (100%)	
		0%	0%	0%	0.89%		
Job dissatisfaction	Yes	7 (23.33%)	11 (36.67%)	4 (13.33%)	8 (26.67%)	30 (100%)	0.045 * ω = 0.188
		17.95%	22.45%	14.81%	7.14%		
	No	32 (16.24%)	38 (19.29%)	23 (11.68%)	104 (52.79%)	197 (100%)	
		82.05%	77.55%	85.19%	92.86%		

* Chi-Square test. ** Kruskal–Wallis H test. B1 is a bruxism axis established in STAB. Data from the study were interpreted according to it. AB = awake bruxism, SB = sleep bruxism. Values in grey are percentages summed by columns for each parameter.

**Table 5:** Orofacial pain, sleep disorder, insomnia, restless feet syndrome and smoking in the study groups (B2 in STAB—concurrent sleep-related consequences and conditions).

Parameter	Value	Possible Bruxism	No Bruxism	Total	*p* *
		115 (50.66%)	112 (49.34%)		
Oro-facial pain in the past month	Never	61 (42.07%)	84 (57.93%)	145 (100%)	<0.0005 ** r = 0.266
		53.04%	75%		
	Rarely	30 (54.55%)	25 (45.45%)	55 (100%)	
		26.09%	22.32%		
	Sometimes	13 (81.25%)	3 (18.75%)	16 (100%)	
		11.3%	2.68%		
	Often	11 (100%)	0 (0%)	11 (100%)	
		9.57%	0%		
	Continuously	0 (0%)	0 (0%)	0 (0%)	
		0%	0%		
Chronic oro-facial pain in the past 6 months	Never	83 (44.86%)	102 (55.14%)	185 (100%)	<0.0005 ** r = 0.257
		72.17%	91.07%		
	Rarely	16 (61.54%)	10 (38.46%)	26 (100%)	
		13.91%	8.93%		
	Sometimes	11 (100%)	0 (0%)	11 (100%)	
		9.57%	0%		
	Often	5 (100%)	0 (0%)	5 (100%)	
		4.35%	0%		
	Continuously	0 (0%)	0 (0%)	0 (0%)	
		0%	0%		
Sleep disorders score	Median values	4.00	2.00	-	<0.0005 ** r = 0.319
	Mean values	3.88 ± 1.97	2.51 ± 2.14		
	CI	[3.51; 4.24]	[2.11; 2.91]		
Insomnia score	Median values	3.00	3.00	-	0.116 ** r = 0.104
	Mean values	2.74 ± 2.16	3.12 ± 1.87		
	CI	[2.34; 3.14]	[2.77; 3.47]		
Restless feet	Yes	26 (70.27%)	11 (29.73%)	37 (100%)	0.009 * ω = 0.173
		22.61%	9.82%		
	No	89 (46.84%)	101 (53.16%)	190 (100%)	
		77.39%	90.18%		
Smoking	Yes	51 (53.68%)	44 (46.32%)	95 (100%)	0.440 * ω = 0.051
		44.35%	39.29%		
	No	64 (48.48%)	68 (51.52%)	132 (100%)	
		55.65%	60.71%		

* Chi-Square test. ** Mann–Whitney U test. Values in grey are percentages summed by columns for each parameter.

**Table 6:** BEWE index in study group—bruxism type defined by questionnaire (227 participants) (A6 in STAB—tooth wear index).

Parameter	Value	AB	SB	AB and SB	No Bruxism	Total	*p*
		39 (17.18%)	49 (21.59%)	27 (11.89%)	112 (49.34%)		
BEWE score	Median values	3.00	3.00	6.00	0.50	-	<0.0005 ** η^2^ = 0.498
	Mean values	2.38 ± 1.69	4.18 ± 2.05	5.30 ± 2.44	0.87 ± 1.23	-	
	CI	[1.84; 2.93]	[3.59; 4.77]	[4.33; 6.26]	[0.64; 1.10]	-	
BEWE category	Risk	17 (18.90%)	32 (35.60%)	14 (15.60%)	27 (30.00%)	137 (100%)	<0.0005 * ω = 0.343
		43.60%	65.30%	51.90%	24.10%		
	No risk	22 (16.10%)	17 (12.40%)	13 (9.50%)	85 (62.00%)	90 (100%)	
		56.40%	34.70%	48.10%	75.90%		

* Chi-Square test. ** Kruskal–Wallis H test. AB = awake bruxism, SB = sleep bruxism. Values in grey are percentages summed by columns for each parameter.

**Table 7:** BEWE index in study group—bruxism type defined by sEMG (48 participants) (A6 in STAB—tooth wear index).

Parameter	Value	AB	SB	AB and SB	No Bruxism	Total	*p*
		39 (17.18%)	49 (21.59%)	27 (11.89%)	112 (49.34%)		
BEWE score	Median values	2.00	4.50	6.00	3.00	-	0.099 ** η^2^ = 0.075
	Mean values	3.27 ± 2.49	4.50 ± 1.56	5.17 ± 1.69	4.31 ± 2.52	-	
	CI	[1.60; 4.95]	[3.50; 5.50]	[4.09; 6.24]	[2.78; 5.84]	-	
BEWE category	Risk	5 (12.50%)	12 (30.0%)	12 (30.0%)	11 (27.50%)	8 (100%)	0.001 * ω = 0.267
		45.50%	100.0%	100.0%	84.60%		
	No risk	6 (75.0%)	0 (0.0%)	0 (0.0%)	2 (25.0%)	40 (100%)	
		54.50%	0.0%	0.0%	15.40%		

* Chi-Square test. ** Kruskal–Wallis H test. AB = awake bruxism, SB = sleep bruxism. Values in grey are percentages summed by columns for each parameter.

**Table 8:** 24 h sEMG indices for study groups in non-instrumental and instrumental allocation of groups.

	Questionnaire and Clinical Exam (Non-Instrumental Allocation of Groups)				sEMG 24 h (Instrumental Allocation of Groups)			
Parameter	AB (n = 12)	SB (n = 12)	SB and AB (n = 12)	No B (n = 12)	AB (n = 11)	SB (n = 12)	SB and AB (n = 12)	No B (n = 13)
	Median	Median	Median	Median	Median	Median	Median	Median
SB-BWI	0.082	0.286	0.281	0.101	0.093	0.423	0.389	0.058
SB-BTI	0.186	0.421	0.355	0.191	0.196	0.576	0.480	0.140
SB-BPI	0.151	0.376	0.331	0.174	0.162	0.525	0.485	0.113
no events/h	1.610	3.485	3.675	2.055	1.980	5.395	4.580	1.740
SB-clenching	6.500	25.000	29.000	9.000	8.000	38.000	33.000	11.000
SB-grinding	2.000	1.000	2.500	1.000	2.000	1.000	3.000	0.000
SB-others	0.000	0.000	0.000	0.000	0.000	0.000	0.000	0.000
S-MWI	0.402	0.515	0.513	0.284	0.303	0.738	0.593	0.282
S-MTI	1.625	1.874	1.852	1.108	1.293	2.767	2.330	1.103
S-MPI	1.212	1.420	1.405	0.813	0.963	2.090	1.771	0.833
AB-BWI	0.453	0.424	0.474	0.364	0.453	0.411	1.037	0.150
AB-BTI	0.867	0.535	0.848	0.540	0.979	0.535	1.334	0.239
AB-BPI	0.719	0.489	0.738	0.470	0.766	0.489	1.235	0.232
AB-clenching	93.500	87.000	61.000	71.500	112.000	76.500	167.000	18.000
AB-grinding	6.000	3.000	9.500	3.000	12.000	2.500	27.500	1.000
AB-others	0.000	0.000	0.000	0.000	1.000	0.000	0.000	0.000
A-MWI	3.236	3.141	3.550	2.560	2.857	3.141	4.051	2.054
A-MTI	14.038	16.583	15.224	11.272	11.272	17.356	15.783	9.413
A-MPI	10.463	12.272	11.408	8.467	8.467	12.618	11.907	6.776
MP	330.500	352.000	325.000	299.000	333.000	348.000	334.000	277.000

AB = awake bruxism, SB = sleep bruxism. BPI = bruxism personal index, BTI= bruxism time index, BWI = bruxism work index. MPI = masseter personal index, MTI = masseter time index, MWI = masseter work index, S = sleep, A = awake, MP = muscular power. Data collected through electromyography obtained information from STAB axis A7.1 (electromyography for sleep bruxism) and A8.2 (electromyography for awake bruxism).

**Table 9:** Diagnostic test accuracy.

Parameter	Sensitivity (%)	Specificity (%)	PPV	NPV
Possible AB	55	84	50	86
Daytime awareness of clenching	55	84	50	86
Possible SB	67	89	67	89
Sounds reported by bed partner	67	89	67	89
Possible SB AB	50	83	50	83
No bruxism	46	83	50	81
BEWE	15	83	25	73

PPV = possible predictive value, NPV = negative predictive value. AB = awake bruxism, SB = sleep bruxism. BEWE = basic erosive wear examination.

## Data Availability

The authors declare that the data of this research are available from the corresponding authors upon reasonable request.
